# Reflecting on Responsible Conduct of Research: A Self Study of a Research-Oriented University Community

**DOI:** 10.1007/s10805-021-09418-0

**Published:** 2021-06-04

**Authors:** Rebecca L Hite, Sungwon Shin, Mellinee Lesley

**Affiliations:** 1 Department of Curriculum and Instruction, STEM Education, Texas Tech University, 3002 18th Street, Box 41071, Lubbock, TX 79409 USA; 2 Department of Curriculum and Instruction, Educational and Instructional Technology, Texas Tech University, 3002 18th Street, Box 41071, Lubbock, TX 79409 USA; 3 Department of Curriculum and Instruction, Language and Literacy Studies, Texas Tech University, 3002 18th Street, Box 41071, Lubbock, TX 79409 USA

**Keywords:** Ethical Dilemmas, Research-Oriented Universities, Responsible Conduct of Research, Self-Study

## Abstract

Research-oriented universities are known for prolific research activity that is often supported by students in faculty-guided research. To maintain ethical standards, universities require on-going training of both faculty and students to ensure Responsible Conduct of Research (RCR). However, previous research has indicated RCR-based training is insufficient to address the ethical dilemmas that are prevalent within academic settings: navigating issues of authorship, modeling relationships between faculty and students, minimization of risk, and adequate informed consent. U.S. universities must explore ways to identify and improve RCR concerns for current (faculty) and future researchers (students). This article reports the findings of a self-study (*N* = 50) of research stakeholders (students and faculty) at a top tier research institution. First, we report on their perceived importance of applying RCR principles. Second, we explore relationships between stakeholder backgrounds (e.g., prior training, field, and position) and how they ranked the degree of ethical concerns in fictitious vignettes that presented different unethical issues university students could encounter when conducting research. Vignette rankings suggested concerns of inappropriate relationships, predatory authorship and IRB violations which were judged as most unethical, which was dissimilar to what sampled researchers reported in practice as the most important RCR elements to understand and adhere to for successful research. Regression models indicated there was no significant relationship between individuals’ vignette ethics scores and backgrounds, affirming previous literature suggesting that training can be ineffectual in shifting researcher judgments of ethical dilemmas. Recommendations for training are discussed.

## Introduction

Responsible Conduct of Research (RCR) is an umbrella term generally defined as engaging in a scientific investigation (research) with ethical purpose and integral practices throughout the process (Shamoo & Resnik, 2009). To accomplish this aim of RCR requires education (training) and awareness (understanding), but perhaps most important, appropriate application of specific professional norms and ethical principles in the performance of all activities related to engaging in the full ecosystem of research. This is to ensure excellence and the public’s trust in scientific research, playing a fundamental component in the preparation (programs) of those who will engage in this endeavor as per the America COMPETES Act of 2007 (GovTrack.us., 2018). Federal intervention to mandate RCR has been the response to public outcry starting from the 1950s (e.g., Nuremberg Trials), into the 1960s (e.g., Thalidomide use in pregnant women), the 1970s (e.g., Tuskegee Syphilis Study) and the 1980s (after publication of the Belmont report in 1978) on case after case of research misconduct (see Rice, 2008). From this, grew specific concerns and a focus on “the way students were trained in the ethical aspects of research” (Steneck & Bulger, 2007, p. 829). Therefore, instruction in research integrity has been a feature of U.S. graduate education in the past 30 years in STEM subjects (National Institutes of Health [NIH], 1989; 1994). In the social sciences, the National Research Act of 1974 paved the way for human subject protections and creation of the modern Institutional Review Board system designed for regulation of human subjects research (Rice, 2008). The RCR generally includes four core areas related to: (1) the human participant and animal subject protections and use of data, (2) responsible publication practices and authorship, (3) ethical practices for peer review, and (4) identifying and disclosing conflicts of interest (Fisher et al., 2009). Yet, despite strategies for teaching RCR elements to students, the incidence of reported cases of research misconduct at the NIH (2016a), similar agencies, and academic settings across the country have been increasing and rampant (Master et al., 2018). Understandably, this has led to lawsuits and loss of funding for persons and institutions who have violated RCR, facilitating a public distrust of research, especially in traditionally vulnerable populations like children and African Americans (Gamble, 1993; Rajakumar et al., 2009). These violations are concerning given that university programs seldom teach the principles and standards of research in a formal way to newly admitted graduate students who have inadequate or non-existent knowledge of RCR, despite implicit expectations of their full participation in or conducting independent research (Plemmons et al., 2006). Moreover, students’ actual exposure to research practices varies across schools and even within a single university’s programs (Heitman et al., 2007). Many universities utilize training provided through the Collaborative Institutional Training Initiative (CITI) Program (2020), whose training modules credential upward to millions of researchers in various RCR-related topics required by thousands of universities and other organizations before they engage in research activities. With more studies occurring within the community, “investigators and study personnel are required by their university to complete training in [both] the ethical and regulatory aspects of human subject’s research” (Yonas et al., 2016, p. 97). Despite these earnest efforts, regulatory or ethical violations of research due to ignorance of or willful disregard for RCR requirements (Fisher et al., 2009) undermine the overall integrity of university-based research (Eisen & Berry, 2002). This has been identified by university-level administration as a clear concern and evident need for the research community (Evola, n.d.).

## Need and Gap for Study

There is a long-held expectation that students receive their RCR training from working with mentors (advisors), or through mentions of RCR throughout their coursework, has done little to stem the problem of RCR (Steneck & Bulger, 2007). Changing demographics of university students (i.e., moving away from full-time on campus toward part-time online work, see Polson, 2003), the increasing complexity of engaging in human-based research (e.g., multi-site trials, calculating risk/benefit analyses, obtaining versus waiving consent, see Porter et al., 2018), compounded by the ongoing challenges of conducting research online (Padala et al., 2020), with rigorous peer review (Denis-Oliveira, 2020), and providing access to remote RCR re/training (Sohrabi et al., 2021) due to COVID-19 interruptions present additional challenges to maintaining student and researchers’ knowledge and skills of RCR. Specifically, the challenge is having researchers apply all RCR principles appropriately and consistently. And as such, apprentice-based approaches in learning how to conduct research ethically have been historically and largely inadequate in conveying and enacting RCR (Folse, 1991; Heitman et al., 2005). This inadequacy suggests that there are further RCR-related issues among faculty and other university research stakeholders worth exploring. For example, Trinkle et al. (2017) found that faculty were able to pressure students into questionable research practices, like egregious honorary authorship, despite their receiving prior RCR training. This finding suggests that variables, beyond receipt of RCR training, influence the culture of RCR adherence on university campuses. Similarly, Kovacs (2017) suggests that academic capital (power and seniority) can translate into predatory authorship, that belies having possession of intellectual capital and erodes the meritocratic foundation of academe. Academic power has nuance beyond faculty and student dichotomies, which is only one of many variables that may influence the application of RCR within a university setting. Other factors like field of study, time at the university, recent activity in conducting research, as well as training, may influence one’s RCR. Therefore, taking a snapshot of RCR understandings and activities among all representative members of a research community, at the campus level, is warranted to garner a preliminary understanding of the broader community of researchers’ perceptions of RCR. This includes how researchers apply RCR in authentic scenarios experienced at a tier 1 research university, and potential relationships between their backgrounds (e.g., prior RCR training, field of study, position at the university).

Thus far, the discussion has centered on internal forces that influence RCR. External forces (and namely funding) are shifting university research priorities toward increased publications (Auranen & Nieminen, 2010) while paying for authorship on publications from grant funds (Sweedler, 2019) or open access to boost article citation counts (Morillo, 2020). There is also a push toward higher quantity but lower quality research outputs (Civera et al., 2020; Laudel, 2006). Funding has now even directed researchers toward specific research topics (Geiger, 1997; Kyvik, 2007) and away from others (Farooq et al., 2020; Laudel & Gläser, 2014). As such, federal agencies like the National Institutes of Health (NIH) and National Science Foundation (NSF) require strict compliance with RCR principles (Steele et al., 2016), which in turn increases the importance of research universities to enforce RCR to maintain their current grants and the ability to apply for future monies. For example, the NIH (2016b) explicitly recommends training on RCR to be held within three weeks of a researcher’s arrival on campus so that they may discuss ethical principles, be exposed to vignettes or real-world scenarios to test their knowledge of RCR practices, and become aware of mentoring and annual review mechanisms. Therefore, we believe that there is an urgent need to gauge the extent to which research stakeholders in universities access and value concepts presented through training, which can significantly mediate or predict task-based transfer (Alliger et al., 1997). We also believe the importance of examining how stakeholders perceive the nature of RCR and what they believe is the best way(s) to address the training of researchers, especially graduate students, on RCR at a research-oriented university.

## Purpose of Study

This study is a partial replication of research conducted by Artino (2007), who explored the relationships between RCR-types of training and researchers’ abilities to navigate ethical dilemmas related to RCR in education-focused research. His research was informed by prior research that suggested formal ethics training had no impact on graduate student researchers’ ability to assess ethical dilemmas (Sales & Folkman, 2000) despite the calls for greater ethics training to address and mitigate issues of research misconduct (Wadman, 2005). This study extends beyond Artino’s study of graduate students in education to the entire university community. We attempted to address the need for universities to explore the outcomes of their RCR education in two ways. First, we explored literature-based findings that apprenticing with faculty does not stem the issues of RCR concerns (Folse, 1991; Heitman et al., 2005), and that training does not equate to RCR compliance (Anyansi-Archibong, 2015; Weyrich & Harvill, 2013). We then explored various aspects of the university research community to understand how to best leverage curricular resources (Kalichman, 2013). However, understanding the underpinnings of their responses to said dilemma requires exploration of how research stakeholders within a research-oriented community identify which tenets of RCR have the greatest importance. Thus, our research-based first step is to explore how researchers ranked the importance of RCR principles. Suppose faculty and students rank certain RCR tenets differently; this may indicate they hold different perspectives on which tenets are relatively more important, despite being held to the same RCR standards. Asymmetrical perceptions between faculty and students warrant further study and additional RCR training or nuanced RCR supports. Our second and third step would be to posit applicable research vignettes in which unethical behavior related to RCR is taking place. Having researchers rate the ethical nature of the vignettes and identify which vignettes they believed would be most likely to occur on campus, would provide a greater understanding of how RCR training is applied within real-world scenarios. The fourth and last step would be to relate stakeholder backgrounds, like prior RCR-related training, a field of study, position, and duration at university, to how they rank the degree of ethical concerns in fictitious vignettes. Any significant relationships may suggest patterns affirming prior research on the inadequacy of ethics training, given that relationships between ethics training courses and research outcomes, remain unclear, and thusly, identified as an area for future research (Steele et al., 2016).

## Research questions:

To explore our four steps of inquiry, we surveyed research stakeholders (students and faculty) who engage in research at a research-oriented university to develop an understanding of how they would:

- 1. Rank the importance of RCR principles from most to least important regarding what all researchers should know and be able to do related to RCR?
- 2. Rate ethical vignettes that apply to campus-related RCR issues?
- 3. Perceive which of the ethical vignettes is most and least likely to occur on campus?

And last, research question 4 asked if there are relationships (correlations) between their ranking of RCR principles and assessments of the vignettes to their (i.e., position at the university, a field of study, time at the university, currently engaging in research, and types of RCR-related training)?

This type of research is important for research-oriented universities given the growing body of literature evidencing relationships between ethics policies and actions to organizational outputs (Bento et al., 2017; Kaptein, 2015; Smith-Crowe et al., 2015). This research can be rationally extended to consider the relationships between RCR policies and training and university level outcomes, potentially adding to current knowledge of how various stakeholders would address different ethical issues university students might encounter when conducting research with faculty.

## Method

We invited members of the research community at a single research-oriented university to participate in a Qualtrics survey, which was sent out weekly from November 29th, 2018 to May 29th, 2019 as an advertisement on the university-wide announcement service. The survey was also sent out via campus email to various sectors of the research community to ensure coverage of survey dissemination. These groups included research services (Office of Research, IRB, IACUC), university-level administrators (president, provost, directors), college-level administrators (deans, associate or vice deans, department chairs), faculty (non and tenure-track, assistant, associate, full), as well as graduate and undergraduate students (online, on-campus, part-time and full time). The survey asked background questions about their position at the university (faculty, student), years of service at the university, prior RCR training records (e.g., CITI, ethics, additional training), and present level of research activity (in the past, currently conducting research, will be conducting research). Survey items were comprised of two sections, ranking their perceived importance of various elements of RCR and assessing ethical issues, which are discussed in detail below. This study was approved by and conducted under the auspices of the University’s IRB board (IRB2018-811).

### Ranking Importance of RCR principles. 

To understand how members of the research-oriented university community valued various principles of RCR, they were asked to rank nine aspects of RCR (in no particular order): (a) understanding and negotiation authorship; (b) modeling and/or exercising appropriate relationships between research team members; (c) engaging in principled human or animal subject research; (d) responsible publication practices; (e) ethical peer review; (f) proposing use of data and responsible data management; (g) identifying and disclosing conflict(s) of interest; (h) understanding IRB or IACUC policies and procedures; and (i) practicing environmental health safety and laboratory safety. Specifically, ranking by the prompt of which are the most important (1st) to least important (9th) among these RCR core concepts for all researchers to know and be able to do. These principles do not include all RCR principles, however, delineate major aspects of RCR (see Fisher et al., 2009; NIH, 2009). Therefore, for analysis, the ranking function was used in Microsoft Excel. After organizing the data of individuals’ rankings to each of the nine RCR attributes, counts were calculated within cells with a specific condition (COUNTIF function) to ascertain how many times a rank (1–9) appeared within each RCR attribute. Then, those values were calculated into total ranks by having each rank value divided by each rank and summed. Final rankings (RANK function) were calculated by taking the total rank value for each RCR attribute, compared to all ranks’ values. This process was repeated by variables of interest: (a) position at the university, (b) field of study, (c) time spent at the university (either more or less than five years), (d) currently engaged in research at the university level, and (e) types of RCR-related training completed (i.e., CITI, ethics, both CITI and ethics, or other additional training).

At the end of this question, respondents were asked to provide any additional information that they believed was missing from the list or RCR aspects that may be more important to certain groups of students (undergraduate, masters, and doctoral) than others. Twenty-one individuals entered a free response, 15 faculty and six students. Their recommendations are presented in the results section as a means to provide visualization to the quantitative data, and thusly were not analyzed using any specific methodology.

### Ethical Vignettes.

Participants were given nine different vignettes that presented an ethical dilemma. These vignettes were sourced from a validated instrument in a published study (see Artino, 2007), which examined the relationship between formal ethics training and assessments of ethical dilemmas. In this study, we presented survey respondents with nine scenarios.The ethnical vignettes are as follows:

1. In the first vignette, “a *typical* data set,” a graduate student raises a concern to his professor that they had described a research data set as *typical*, in which it was not assessed (or known) as such, for an accepted conference abstract. The professor dismisses their concern, so the respondent is asked to assess the ethics of the professor’s response.
2. The second vignette, “remove outliers,” describes the actions of a doctoral student who has unilaterally decided to remove all outliers from their dissertation data. Respondents are tasked with determining the ethics of such actions.
3. The third vignette, “no recommendation,” describes an outstanding recent graduate who communicates to his advisor that he would like a letter of recommendation for a position in the industry. The advisor is dismayed at this news, expressing his disappointment regarding the graduate student’s decision. The advisor also states to the graduate student that his recommendation will not be as strong since he can only speak to the candidate’s potential in an academic rather than industrial setting. The respondent is asked to assess the ethics of the advisor’s behavior.
4. The fourth vignette, “no IRB needed,” describes a conversation between a professor and their teaching assistant (TA) regarding the administration of an anonymous survey about student’s study habits and how their habits relate to attitudes about the course. When the TA asks about submitting an IRB proposal, the professor indicates they will only use the results for course improvements unless there is something publishable. And if publishable, the professor would, at that time, seek and obtain IRB approval. The respondents are asked to determine the ethics of the professor of record’s actions in this scenario.
5. The fifth vignette, “two publications from one,” regards the deliberation of a recent graduate parsing his dissertation data into two publications. Despite the data being intertwined under a single research question and theoretical framework, the new graduate decides to forge ahead with separating the data into two manuscripts. Respondents considered the ethical nature of this decision.
6. The sixth vignette, “authorship for all,” describes the perspective of a highly successful professor with a large research lab and publication record. For any manuscript produced from the lab, the professor ensures each lab member’s name is on it, even if they did small amounts of data collection or data entry. Respondents consider the ethics of this approach to authorship.
7. The seventh vignette, “stay in the study,” describes the actions of a graduate research assistant (GRA) coaxing a student to remain in a research study after he expressed a desire to withdraw from the experiment. The graduate student does not force the student to remain in the study rather reminds them about the drawing (e.g., potential participant support), and how annoyed the Principal Investigator or PI (the graduate student’s employer and the student’s professor of record) can get when students leave studies. Respondents assess the ethics of the GRA.
8. The eighth vignette, “make me the second author,” describes the negotiation over manuscripts between a dissertation chair and his advisee on dissertation products. When the chair provided edits to the article, he insisted on being added as the second author since he is well known in the field and the journal. The student acquiesces and submits the manuscript with her chair as the second author. The respondent considered the ethical behavior of this request and subsequent action.
9. The ninth and final vignette, “inappropriate relationships,” describes the actions of a GRA and their PI outside of the laboratory. During a break from work, the GRA and PI share a meal and engage in informal (non-work) conversation. The GRA asks the PI to walk her to her apartment and then, invites the PI inside for a night cap. Respondents were tasked with assessing the PI’s behavior in the scenario.

To record their thinking on these ethical scenarios, we asked participants to rate each of the nine vignettes using a seven-point Likert scale, a value of one assessed as “extremely unethical” and a value of seven assessed as “not an ethical issue”. This rating was to prompt the reader to consider first, to what degree this issue involved ethics (if not, they may immediately select not an ethical issue) and second, if deemed to involve ethics, to consider the degree of the un/ethical behavior (on the Likert scale).

Vignettes are powerful tools of qualitative research by allowing participants to envision situations in a real-world context as well as useful for sensitive topics that may be hard to discuss through interviews or other data collection methods (Barter & Renold, 1999). By providing contextual applications of ethical dilemmas, especially those situated to difficult situations that one could encounter within higher education, provides greater understanding of how individuals would authentically enact their RCR training principles. Given that these vignettes are *anchored* in higher education, any stakeholder (regardless of research field, training or position within the university) can envision and assess the presented situation, which is why anchored vignettes are useful in identifying differences even among disparate groups (King & Wand, 2007). Notably, this identification does not provide any explanation of differences in groups; therefore, further analyses are required (like regression, which was used in this study) to explore relationships among groups (Grol-Prokopczyk et al., 2011). Last, they were asked which of the scenarios would be most likely and least likely to occur on campus, and given an opportunity to justify their response with a short answer. Therefore, for analysis, Likert values were summed by means, and evaluated further by standard deviation, checking for skewness and kurtosis by scenario per the analysis conducted by Artino (2007). Also replicated from Artino’s analyses, five regression models were developed in STATA (2020) to explore possible significance of vignette responses by vignette means scores and types of RCR-related training, position (faculty and students), years at university (more or less than five years), currently conducting research (yes or no), and field of study (social sciences and STEM). T-tests were conducted as a means of hypothesis testing on regression coefficients obtained in each of the linear regression models. At the end of this question, respondents were again invited to provide any additional information on their personal research experiences related to RCR as an open-ended response. Eight respondents (5 faculty and 3 students) provided a response. Quotations are presented in the results section as a means to provide visualization to responses only and are not part of the analysis.

### **Participants.**

Participants for the research study included a random sampling of 50 individuals who identified as researchers at one research-oriented university in the southwestern United States. Among the 50, half were faculty (*n* = 25) and nearly half were students (*n* = 24). Among the 25 faculty members sampled, they included tenure track full professors (*n* = 9, 36% of faculty sample), tenure track associate professors (*n* = 4, 16%), college level administrators (*n* = 4, 16%), research services (*n* = 3, 12%), tenure track assistant professors (*n* = 2, 8%), non-tenure track faculty (*n* = 2, 8%), and a university level administrator (*n* = 1, 4%). Among the 24 students, this was mainly undergraduate students (*n* = 11, 46% of student sample), followed by doctoral level graduate students (*n* = 10, 42%), and masters level graduate students (*n* = 3, 12%). One person did not provide position information. Fields of those sampled varied, with almost half sourced from Arts and Social Sciences (*n* = 24, 48% of entire sample), followed by the STEM fields (*n* = 14, 28%), and the Professional sector consisting of the Law, Business, and Medical schools respectively (*n* = 4, 8%). Six individuals (12%) indicated a field did not apply as they were not in a position of research, and two persons (4%) declined to identify their specific field. Regarding how long they have been at the university, 15 (30%) have been at TTU for less than two years, 14 (28%) had been at TTU for 3–5 years, 6 (12%) 5–10 years, 11 (22%) between 10 and 20 years, and four (8%) longer than 20 years, with one person who chose to not respond. To adhere to the structure and recommendations of the IRB, as it was not a part of the research questions, no gender or racial/ethnic identifiers were collected as a part of the survey procedure.

Among the sample, thirty-three participants (68%) indicated they were presently conducting research, six (12%) indicated they had conducted research in the past, three (6%) indicated they have not yet conducted research, but plan to, seven (14%) stated they have never conducted research and do not plan to do so in the future, and one person did not respond to the question; All of these respondents identified as undergraduate students. Notably, undergraduate students receive RCR training as inculcation to the university culture of research, even in their first year, so they remained in the study (Evola, n.d.). Of those who were actively engaged in research, twenty-five participants (47%) stated they are actively conducting research that has been reviewed and approved by the University’s IRB. When asked how many, 16 of these participants provided a response, of approximately 3.91 (as mean, median and mode of 2) active research studies with approved IRBs. Nine participants (17%) were writing proposals or had IRBs under review. When asked how many, seven of these participants provided a response, holding a Mean of 2.29 (Median of 2 and Mode of 2) of IRBs in development and/or under IRB review. The remaining nineteen (36%) indicated that their research was not applicable or eligible for IRB review. Regarding training in RCR, overall, nine (18%) had only completed CITI or NIH training, 12 (24%) had only completed ethics training, 16 (32%) had completed both CITI/NIH training and ethics training, 12 (25%) indicated they had no completely any RCR-related training, and one person did not respond to this question (2%). Of those who had CITI/NIH or ethics training, nineteen participants indicated they had sought and completed additional training provided by the university on RCR principles.

## Results

The following sections describe the results of ranked importance of RCR principles from most to least important concerning what all researchers should know and be able to do at a research-oriented university (research question one), the rating of ethical vignettes that applied to campus-related RCR issues (research question two), perceptions of which ethical vignettes was most and least likely to occur on campus (research question three), and relationships between stakeholders’ backgrounds (i.e., position at the university, field of study, time at the university, currently engaging in research, and types of RCR-related training) and their rankings of RCR principles and ethics vignette scores (research question four).

## Results of Ranking RCR Principles

To explore rankings described in research question one, Tables 1, 2, and 3 show the relative rankings of nine RCR principles by all participants by the importance of what researchers should know and be able to do. Each table contains *all participants* as a benchmark rank (in bold), which were disaggregated by subgroups of position at the university and field of study (Table 1), time and research activity at the university level (Table 2), and types of RCR-related training completed (Table 3). Graduated shading for the top three ranks of each group was used to illuminate differences among group rankings. The *all participant* trend indicated that proper use of data and responsible data management held the first rank as most important (*n* = 329), followed by engaging in principled human and animal subject research (*n* = 316), and third responsible publication practices (*n* = 254). Ethical peer review was fourth (*n* = 233), followed closely by understanding IRB or IACUC policies and procedures (*n* = 232) and identifying and disclosing conflicts of interest (*n* = 231). The bottom of the ranks was modeling and/or exercising appropriate relationships between research team members (*n* = 219), understanding and negotiating authorship (*n* = 219), and last with practicing environmental health safety (EHS) and laboratory safety (*n* = 217).

**Table 1:** Relative Rankings of Nine RCR Principles by Importance (1 being most and 9 being least) by All Participants, Position at the University, and Field of Study (Faculty or Student, Social Sciences or STEM with Professional Fields)

	All Participants (*N* = 50)		Faculty (*n* = 25)		Student (*n* = 24)		Social Sciences Fields (*n* = 24)		STEM & Professional Fields (*n* = 4)	
	Rank	Totals	Rank	Totals	Rank	Totals	Rank	Totals	Rank	Totals
Data Management	1	329	2	170	1	155	1	163	2	155
Human & Animal Research	2	316	1	176	2	132	3	127	1	172
Publication Practices	3	254	3	133	6	112	5*	121	4	118
Peer Review	4	233	5	111	5	117	2	128	8	95
IRB/IACUC Procedures	5	232	4	119	8	107	8*	96	3	128
Conflict(s) of Interest	6	231	7*	103	3	125	5*	121	7	104
Appropriate Relationships	7*	219	6	110	7	108	7	104	6	106
Authorship	7*	219	7*	103	9	101	8*	96	5	112
EHS and Lab Safety	9	217	9	92	4	123	4	124	9	90

*Represents a tied total and tied rank

**Table 2:** Relative Rankings of Nine RCR Principles by Importance (1 being most and 9 being least) by All Participants, Time, and Research Activity at the University (More or Less than 5 years, Currently Conducting or Not Conducting Research)

	All Participants (*N* = 50)		Less than Five Years at University (*n* = 28)		More than Five Years at University (*n* = 21)		Currently Conducting Research (*n* = 33)		Not Currently Conducting Research (*n* = 17)	
	Rank	Totals	Rank	Totals	Rank	Totals	Rank	Totals	Rank	Totals
Data Management	1	329	1	181	1	144	1	231	1	98
Human & Animal Research	2	316	2	172	2	136	2	220	2	96
Publication Practices	3	254	3	144	4	101	3	181	8	73
Peer Review	4	233	5	131	6	97	5	149	5	84
IRB/IACUC Procedures	5	232	8	123	3	103	7	142	4	90
Conflict(s) of Interest	6	231	6	129	5	99	4	152	7	79
Appropriate Relationships	7*	219	4	133	9	85	5	149	9	70
Authorship	7*	219	7	124	8	88	8	138	6	81
EHS and Lab Safety	9	217	8	123	7	92	9	123	3	94

*Represents a tied total and tied rank

**Table 3:** Relative Rankings of Nine RCR Principles by Importance (1 being most and 9 being least) by All Participants and Types of RCR-related Training (CITI, Ethics, Both, and Other)

	All Participants (*N* = 50)		CITI and NIH Training Only (*n* = 9)		Ethics Training Only (*n* = 12)		CITI/NIH and Ethics Training (*n* = 16)		No RCR-Related Training (*n* = 12)	
	Rank	Totals	Rank	Totals	Rank	Totals	Rank	Totals	Rank	Totals
Data Management	1	329	2	57	2	76	2	112	1	76
Human & Animal Research	2	316	1	60	7*	53	1	121	2	73
Publication Practices	3	254	4	46	3	61	3	90	6	54
Peer Review	4	233	6	39	5	57	7	66	3	67
IRB/IACUC Procedures	5	232	3	47	9	45	4	82	7	53
Conflict(s) of Interest	6	231	9	35	3	61	7	66	4	62
Appropriate Relationships	7*	219	5	43	1	79	9	48	9	47
Authorship	7*	219	6*	39	7*	53	5	68	5	58
EHS and Lab Safety	9	217	6*	39	6	55	6	67	8	50

*Represents a tied total and tied rank

Table 1 provides the rankings of all participants by faculty ranks and student ranks as well as ranks from individuals in the social sciences and ranks from individuals in STEM and professional fields (e.g., medicine, business). Faculty rankings largely mirrored the all participant trend, whereas students ranked conflict of interest third. Notably, students also had reverse rankings of IRB/IACUC (8th, *n* = 107) and EHS and lab safety (4th, *n* = 123), compared to faculty and the overall (all participant) trend. Individuals in the social sciences and STEM plus professional fields had similar rankings to the entire group, with social sciences valuing ethical peer review higher (2nd, *n* = 128), and STEM ranking IRB/IACUC higher (3rd, *n* = 128) compared to overall rankings. Interestingly, social science fields had a higher ranking of EHS and lab safety (4th, *n* = 124) than compared to the STEM and professional group (9th, *n* = 90). Peer review among the STEM and professional group was ranked appreciably lower (8th, *n* = 95) compared to that of the overall rankings.

**Fig. 1:**
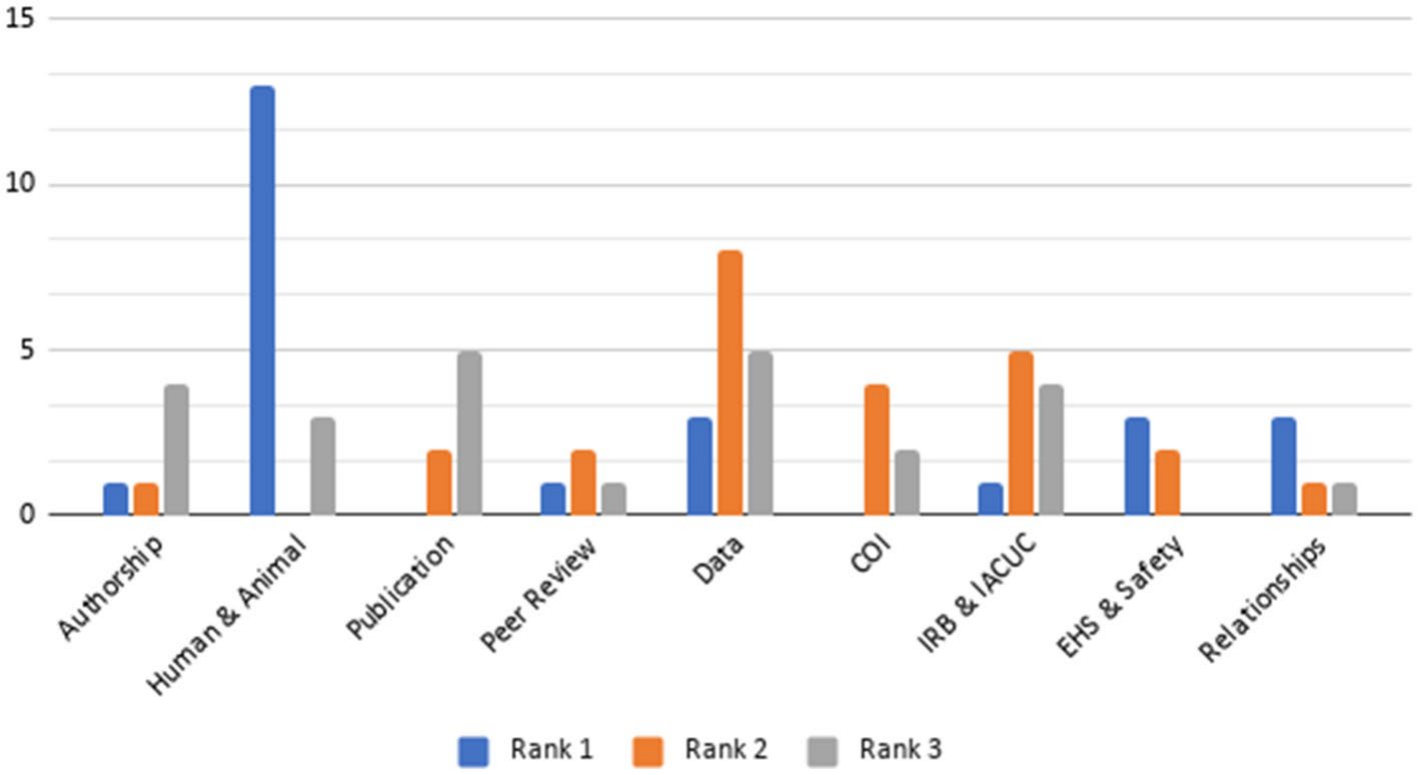
Bar Graph of the Top Three Most Highly Ranked RCR Principles from Sampled Faculty

**Fig. 2:**
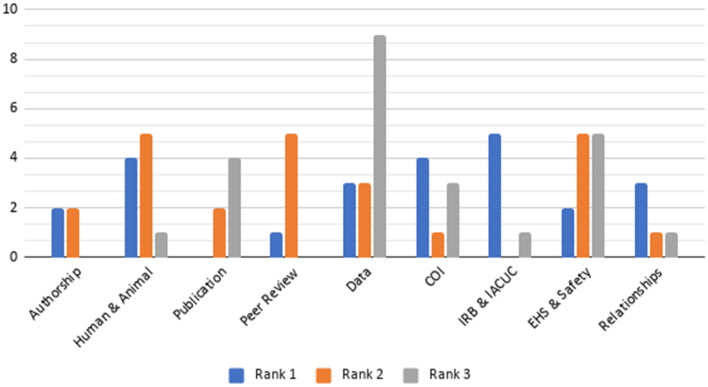
Bar Graph of the Top Three Most Highly Ranked RCR Principles from Sampled Students

Table 1 suggests some similarities between faculty’s and students’ rankings of RCR principles. Figures 1 and 2 provide a graphical illustration of these differences using just the top three ranks of sampled faculty (Fig. 1) and students (Fig. 2). Among faculty top ranks in Fig. 1, we can see consensus on the importance of human and animal subject care, followed by data and policy compliance (i.e., IRB, IACUC, conflict of interest (COI), EHS, and safety).

Among the top three ranks of sampled students, shown in Fig. 2, policy compliance was ranked highest, and data was a common third rank. Notably, students ranked authorship, peer review, and relationships among the top three ranks more than faculty.

Table 2 shows the relative rankings of nine RCR principles by all participants and by subgroups of their time at the university (more or less than five years) and if the respondent reported whether or not they are currently conducting research. Individuals with fewer than five years at the university had similar top three rankings to overall rankings, with a notable exception of ranking modeling or exercising appropriate relationships 4th (*n* = 133) versus 7th (*n* = 219) overall. Also, they ranked safety and IRB/IACUC procedures similarly at the bottom of the RCR principles in importance (tied for 8th, *n* = 123). Individuals with more than five years at the university ranked IRB/IACUC as the third most important (*n* = 103) compared to 5th (*n* = 32) overall. For individuals who reported they were currently conducting research, the top three rankings were again similar to overall rankings, with conflicts of interest holding a slightly higher 4th rank (*n* = 152) compared to 6th (*n* = 231) overall. Interestingly, those conducting research did not highly rank the ruling or compliance based bodies of research, being IRB/IACUC (7th, *n* = 142) and EHS and lab safety (9th, *n* = 123). Individuals not currently conducting research ranked EHS and lab safety much higher (3rd, *n* = 94) than overall (9th, *n* = 217), and ranked understanding and negotiating authorship (6th, *n* = 81), responsible publication practices (8th, *n* = 73), and appropriate relationships (9th, *n* = 70) lower as compared to overall.

Table 3 shows the relative rankings of nine RCR principles by all participants and then by subgroups of training completed related to RCR. Individuals who had only CITI or NIH training ranked IRB/IACUC procedures higher (3rd, *n* = 47), as well as publication practices (4th, *n* = 46) and appropriate relationships (5th, *n* = 43) than overall rankings. Individuals who had only ethics training ranked modeling and/or exercising appropriate relationships between research team members as most important (1st, *n* = 79), which was ranked 7th (*n* = 219) overall. Also, this group indicated that identifying and disclosing conflicts of interest was 3rd most important (*n* = 61) and ranked human and animal research much lower (7th, *n* = 53) compared to 2nd (*n* = 316) overall. Whereas the individuals who reported to having both CITI or NIH and ethics training had similar rankings to overall, with exceptions of ranking ethical peer review (7th, *n* = 66) and appropriate relationships (9th, *n* = 48) much lower than overall rankings (4th, *n* = 233 and 7th, *n* = 219, respectively). Individuals sampled who reported no RCR-related training had similar rankings to those who had both types of RCR training, except for understanding IRB/IACUC procedures, which received a low ranking (7th, *n* = 53), and conflict of interest (4th, *n* = 62) and authorship (5th, *n* = 58) which received higher rankings of importance.

Twenty-one individuals provided an open-ended response to the question of which RCR was missing; their position and field are provided in parentheses following their quote or recommendation. Issues of non-compliance, like having explicit “protocols for handling colleagues who engage in research misconduct” (College level administrator in Arts & Sciences), clear guidance for understanding “plagiarism” (non-tenure track faculty in Arts & Sciences), for the “ethical use of funds, including PI support/salary and documentation of that effort” (full professor in STEM) were mentioned. Others indicated more nuanced aspects of RCR were needed, that students should understand “methods” and “accurate data analysis and reporting” (two associate professors in Arts & Sciences) as well as the “IRB process and approval, especially when working with vulnerable populations” (assistant professor in Arts & Sciences). Last, there was a suggestion that reflected the changing nature of publication like helping students (and faculty) avoid “predatory publishers” (full professor in STEM). This was echoed by a response from a biomedical graduate student, saying they wanted a better “understanding of journal rankings, publication fees, and open access,” suggesting a need for broader guidance in this growing area of academe. In regard to student groups who may need more training in one RCR area than the other, eight respondents stated undergraduates need more training in the basics of research (*n* = 3), with specific training for engaging in appropriate relationships among team members (*n* = 3), and understanding conflicts of interest (*n* = 2). In regard to RCR training for masters and doctoral students, seven respondents indicated that more emphasis should be placed on graduate-level advanced safety (*n* = 3) and publication-related training (*n* = 4). The six remaining respondents said there should be no differences between the levels (undergraduate, masters and doctoral) students at research universities.

## Results of Ranking Ethical Vignettes

Table 4 displays descriptive statistics of mean, standard deviation, skewness and kurtosis for the nine ethics ratings collected in the study to address research question two. Skewness values suggest fairly symmetrical data for vignettes 7, 8, and 9, with moderate skewness for vignettes 1, 2, 3, 4, and 6. No IRB needed is highly skewed (i.e., a value greater than one). Kurtosis values suggest the distribution is *mesokurtic*, or similar to the normal distribution with minimal probability for outliers. Using mean scores (per Artino, 2007), each vignette was ranked in order from most (1st) to least (9th) unethical.

**Table 4:** Descriptive Statistics for Each of the Nine Vignettes and the Mean Ethics Rating from Most Unethical (Highest Rank Order) to Least Unethical (Lowest Rank Order)

Rank Order (by Mean)	Vignette Number and Description	N	Mean	SD	Skewness	Kurtosis
1	#8—Make Me 2^nd^ Author	50	2.40	1.41	0.95375	0.692151
2	#7—Stay in the Study	50	2.60	1.73	0.981157	0.41153
3	#2—Remove Outliers	50	2.68	1.74	0.891077	-0.174318
4	#3—No Recommendation	50	2.72	1.82	0.860582	-0.392699
5	#4—No IRB Needed	49	2.76	1.70	1.04487	0.232199
6	#9—Inappropriate Relationships*	50	2.82	1.95	0.765373	-0.64923
7	#1 – A Typical Data Set	50	3.78	1.55	0.116281	-0.593162
8	#6—Authorship for All	49	3.92	1.91	0.264007	-1.037695
9	#5—Two Publications from One	50	4.06	1.89	0.295179	-1.20457
-	Mean Ethics Rating	9	3.08	0.75	-0.01161	0.058896

Seven Point Likert Scale: 1, extremely unethical to 7, not an ethical issue

*Inappropriate relationships describes a romantic relationship between a student and professor

Vignette 9 was Vignette 9a from Artino (2007); Vignette 9b was not employed in this study

From the analysis, eight of the nine vignettes (i.e., 1, 2, 3, 4, 6, 7, 8, and 9) had means below the midpoint (i.e., < 4.0) of the seven-point Likert rating scale and were all positively skewed. Of these eight vignettes, the scenario in vignette 8 (make me second author), was rated as the *most* unethical, with a mean score of 2.40 (*SD* = 1.41). Other unethically ranked scenarios were vignette 7 (stay in study), vignette 2 (remove outliers), vignette 3 (no recommendation), vignette 4 (No IRB needed), and 9 (inappropriate relationship), with mean scores of 2.60 (*SD* = 1.73), 2.68 (*SD* = 1.73), 2.68 (*SD* = 1.74), 2.72 (*SD* = 1.82), 2.76 (*SD* = 1.70), and 2.82 (*SD* = 1.95) respectively. The remaining three vignettes, closest to the mid-point of the rating scale (i.e., mean score of 4) were vignette 1 (typical data set), vignette 6 (authorship for all), and vignette 5 (two publications from one) with mean scores of 3.79 (*SD* = 1.55), 3.92 (*SD* = 1.91), and 4.06 (*SD* = 1.89), respectively.

## Occurrence on Campus

**Fig. 3:**
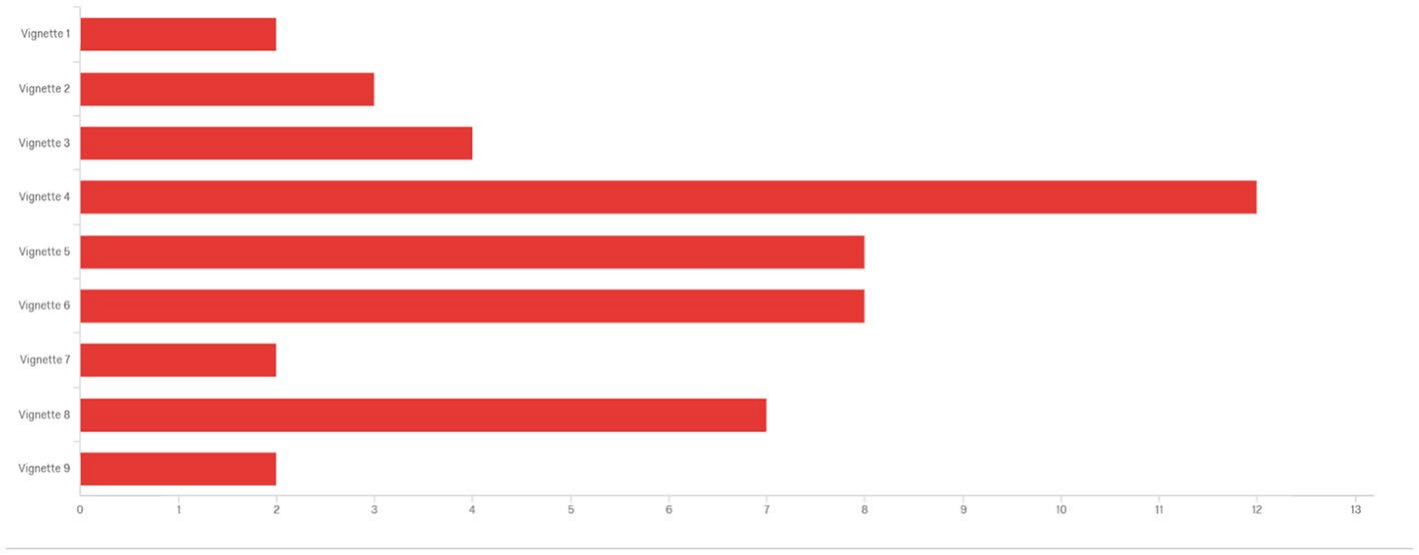
Bar Graph Comparing the Most Common or Likely Ethical Dilemma to Occur on Campus of Vignettes 1 (Top) to 9 (Bottom)

**Fig. 4:**
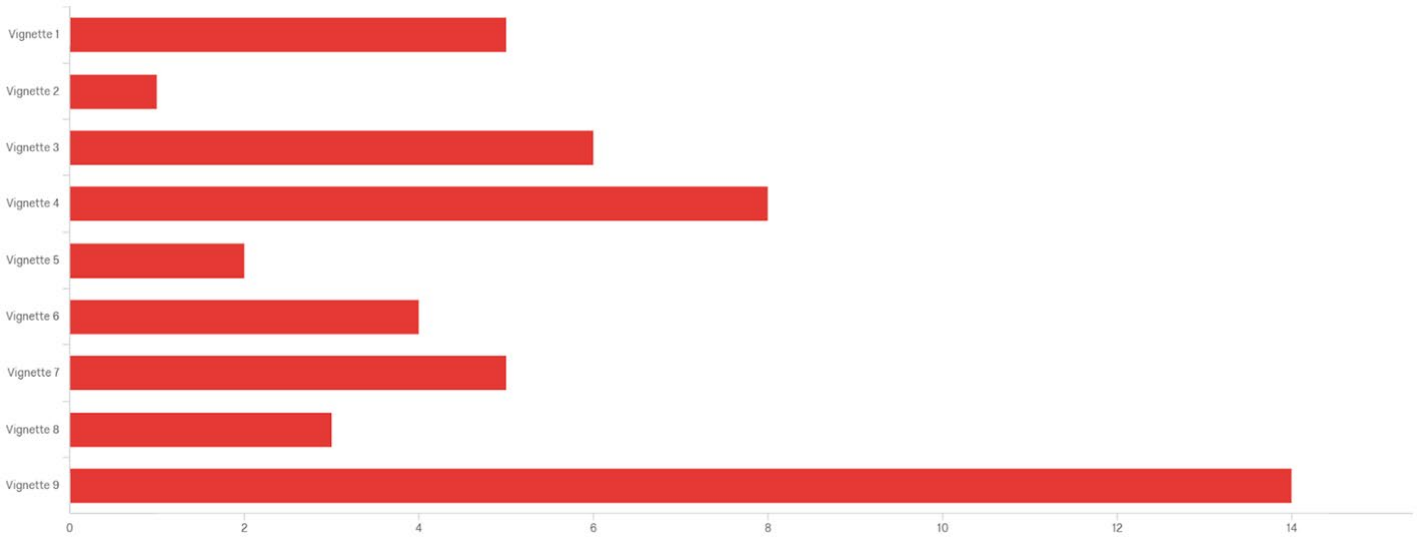
Bar Graph Comparing the Least Common or Unlikely Ethical Dilemma to Occur on Campus of Vignettes 1 (Top) to 9 (Bottom)

Participants were asked which among the nine presented scenarios were the most common and likely to occur on campus (Fig. 3) as well as which of the nine presented scenarios were the least common or most unlikely to occur on campus (Fig. 4) to address research question three.

Figure 4 indicates the vignette that respondents thought was most likely (*n* = 12) was vignette 4 (no IRB needed) followed by a tie for second most likely (*n* = 8) with vignettes 5 (two publications from one) and 6 (authorship for all). Third most likely (*n* = 7) was vignette 8 (make me second author). Whereas in Fig. 3, the least likely to occur (*n* = 14) was vignette 9 (inappropriate interaction), followed distantly (*n* = 8) by vignette 4 (no IRB needed) and vignette 3 (no recommendation, *n* = 6).

## Relationship among Participant Variables and Ethics Vignettes

To review relationships among vignettes and variables to address research question four, we ran five regressions models to ascertain how well various independent variables (background of participants) predicted their dependent variables (Likert or numerical response to each ethical vignette). The first model explored the ability to predict Likert responses to ethical dilemmas by amount of training reported. An insignificant regression equation was found (F(3, 45) = 1.40, *p* > 0.05), with an R^2^ of 0.09. The second model assessed Likert responses by position at the university, where an insignificant regression equation was found (F(1, 47) = 2.31, *p* > 0.05), with an R^2^ of 0.05. A third model examined length at the university to responses, where an insignificant regression equation was found (F(1, 47) = 0.85, *p* > 0.05), with an R^2^ of 0.02. A fourth model of area of research among the sciences to responses produced an insignificant regression equation of (F(1, 46) = 0.02, *p* > 0.05), with an R^2^ of 0.0004. The last model that examined years at the university to responses produced an insignificant regression equation of (F(1, 47) = 0.85, *p* > 0.05), with an R^2^ of 0.177. T-test analyses of each model were also found to be insignificant, affirming all non-significant results from each regression model.

Among the eight individuals who provided additional information on their experiences with ethical dilemmas on campus, they described deliberate and unintentional concerns related to RCR principles. Their position at the university and field followed their contribution in parentheses. First, two respondents described individuals not following ethical rules deliberately where “the major ethical issues I see with research at [this research intitution] involve intellectual property and authorship issues. I have seen many examples of faculty stealing other faculty member's research, writing, and ideas” (college level administrator, Arts & Sciences) and there are “predatory professors (typically much older, white males) prey on tenure track faculty and graduate students” for their publications (assistant professor, Arts & Sciences). The latter behavior is problematic as it leads to the inculcation of students into an erroneous system of authorship. For example, a master’s student in biomedicine:

From my own experience, I would expect that students conducting research likely know little about authorship guidelines…Either the students and PI assume the PI is responsible for it, or the PI forgets to teach what's likely become second nature to them.

In terms of training, two individuals reported how research services were quite successful in helping them “learn something new [where their] passion for ethical research was infectious” (doctoral student, biomedicine). Further, the “IRB has dramatically improved in recent years, becoming a useful resource and authority to our research” (university-level administrator, STEM and Biomedical sciences). This suggest that the resources on campus are providing a great service, yet are not reaching all students. A doctoral student in biomedicine wrote,

I am surprised in the scenarios mentioned in the survey and I am totally lost on what RCR entails and what constitutes ethical or unethical practices in research...In my honest opinion I feel these vital elements are NOT taught to graduate students or NOT enough publicity is given to workshops related to this subject matter. It's sympathetic because majority of PhD students are interested in taking up jobs in academia and therefore may pose a challenge to their career…I now feel empty on not knowing enough on ethical research bearing in mind that I will graduate very soon and would like to venture into academia jobs.

This comment is further corroborated by a faculty respondent who said, “in my position overseeing an academic support center [in Arts & Sciences], my staff and I see RCR issues (primarily plagiarism, but occasionally authorship questions and other issues) on a near-daily basis. I think there are widespread, fundamental misunderstandings about RCR among both graduate students and faculty.” A second research service staff member in biomedicine echoed that “I feel that most of these scenarios are very likely to occur at [this research institution]. Students often do not understand all of the responsibilities that are associated with a research career.”

## Discussion

This study sought to examine the perceptions of stakeholders at a research-oriented university of RCR in their research lives, by asking them to rank the importance of those principles, rate ethical scenarios (vignettes) that relate to RCR, and predict which are most and least likely to occur on campus. Position at the university, field of study, time spent at the university, currently engaging in research, and types of RCR-related training completed were regressed to illuminate possible relationships between researcher backgrounds and perceptions of unethical behavior. In regard to importance, all persons sampled (*N* = 50) ranked highly attributes that related to research culture (e.g., data, research, publications and procedures, except for EHS and lab safety), whereas intrapersonal assessments and interpersonal interactions (e.g., conflicts of interests, relationships, and authorship) were ranked as less important. There were only modest differences in rankings between faculty and students, largely diverging around the importance of interpersonal interactions (see Fig. 1 and 2). This finding may suggest that RCR training on campuses tends to address the more standardized aspects of research, being mostly focused on compliance. Thus, personal interactions among researchers are not as emphasized or discussed in RCR training, since they are not federally regulated. Perceptions and practice may reflect training, meaning that under-emphasized aspects of RCR in training are being reflected in researchers’ survey responses. In regard to ethics in practice (and not training), “most of the time, what is being measured, and consequently managed, is *reactions*, rather than *behavior and decision making*” (Steele et al., 2016, p. 334), which may influence how research stakeholders perceive their relative importance. This finding may help support the recent trend of recruiting Research Ethics Consultants (RECs) as supports to provide advice and recommendations on specific issues that arise in research (Master et al., 2018; Porter et al., 2018) related to both compliance and inter-personal ethical issues.

Field-based differences between STEM and social sciences may relate to what extent these scenarios arise in their respective disciplines (Table 1). For example, a social scientist may not use animal models just as a bench scientist may not engage with human subjects, so the importance of understanding those respective procedures may not be viewed as universally important. Similarly, students may have identified the importance of lab safety rules because they are actively learning those rules as novice researchers, compared to faculty who adhere to said rules but are more concerned with publication and peer review as those are more relevant to their current professional lives. The differential finding concerning the importance of ethical peer review is interesting, perhaps due to biases in social science publication; that is, manuscripts with null findings are more often rejected in the positivist-focused social sciences than the STEM disciplines (Franco et al., 2014). In this sense, social science researchers may draw more importance toward understanding and mitigating this bias.

In examining the amount of time spent at the university, those with fewer than five years and those who were actively conducting research amplified the importance of exercising appropriate relationships (Table 2). Those who have less time at the university do not necessarily represent all students, as they can also include tenure-track faculty and graduate students, many of whom are also actively conducting research to meet tenure guidelines or graduation requirements. Quotations supported the importance of being able to work collaboratively and productively within teams among those faculty and students actively conducting research, to not only avoid predacious misuse of time and efforts on research (as described by faculty), but also to better understand the “rules of the game” in regards to academe and improve the likelihood of being successful in academic futures (as described by the students). This affirms previous research indicating that researchers and students receive training in RCR, yet continue to express a desire for more information and training (Ateudjieu et al., 2010; Folse, 1991). However, Table 3 indicated that rankings were not drastically different among those with a great deal of RCR-related training and those who had none. Therefore, future training (desired by the research community) should be differentiated to meet their needs on specific or desired information on principles and processes of RCR, and ongoing access to on-demand or personally curated resources for continued professional learning (Lotto, 2018).

One of the most intriguing findings of the study emerged from three contradictory rankings of RCR principles. The first inconsistency was found in the ranking of the vignettes (Table 4) to which ethical dilemmas would be most un/likely occur on campus. The most likely ethical concern (Fig. 3) was to circumvent IRB rules, followed by issues of publication and authorship. Yet, the least likely to occur (Fig. 4) were inappropriate interactions and no IRB needed, respectively. This finding is intriguing since inappropriate interactions were deemed a median ethical concern (6th overall in Table 4), yet seen as having the greatest importance (ranked 1st in Table 3) among those who received ethics training. The second finding was the asymmetrical identification of not seeking IRB as both likely and unlikely to occur on campus. This finding suggests a generalized level of inexperience with IRB to understand part and parcel of human subject protections. Third, an interpersonal issue on authorship was seen as the most unethical scenario (Table 4), yet ranked low in importance among the nine RCR principles in Tables 1, 2, and 3. (The exception is shown in the STEM and professional fields ranking it the highest at fifth place, as seen in Table 1.) Given there were no significant relationships among stakeholder backgrounds and responses to ethical dilemmas, the findings indicate all research stakeholders could benefit from expanded training in the areas of IRB as well as negotiating authorship agreements and developing appropriate research relationships.

## Conclusion

This study provides our university and other research-oriented universities a greater understanding of how individuals rate the degree of ethical dilemmas presented through vignettes that describe major aspects of RCR. This self-study highlights the subtle and complex challenges research ethics committees increasingly need to consider by critiquing the view that “training” in itself can resolve individual shortcomings and bolster ethical review capacity. These cogent and bold findings contribute to the field much-needed evidence for improving and supporting existing (and new) supports for RCR and research review committees, whose work is becoming ever more burdensome and demanding. Training should not be viewed as a one-time panacea, rather a useful tool for ongoing dialogue and evolution of ethnical thinking. Research has found university students who enrolled in courses specific just to RCR when surveyed “noted that courses were useful in preparing them to recognize, avoid, and respond to research misconduct” (Plemmons et al., 2006, p. 571). Further, ethical training should be infused throughout the university curriculum and supported at the programmatic, departmental, and college levels (Folse, 1991), which may help address asymmetrical findings of interpersonal concerns of authorship and relationships that arose in this study. Notably, this study did not collect demographic data, such as gender or racial/ethnic identity. Polling for these demographic factors may provide useful contextual information in regard to how demographic groups view ethical priorities differently or illuminate power dynamics that may enable unethical research practices (interactions between faculty less-marginalized and students who are more-marginalized as an example). Future research will explore how novel forms of training will address RCR and mitigate unethical issues that may present itself in our campus community.

## Disclosure Statement

This is to acknowledge there is no financial interest or benefit that has arisen from the direct applications of this research.

## Data Availability

Data and materials of survey can be made available upon request.

## Code Availability

Code availability is not applicable to this research study.
